# Isolation and Molecular Characterization of Banna Virus from Mosquitoes, Vietnam

**DOI:** 10.3201/eid1408.080100

**Published:** 2008-08

**Authors:** Takeshi Nabeshima, Phan Thi Nga, Posadas Guillermo, Maria del Carmen Parquet, Fuxun Yu, Nguyen Thanh Thuy, Bui Minh Trang, Nguyen Tran Hien, Vu Sinh Nam, Shingo Inoue, Futoshi Hasebe, Kouichi Morita

**Affiliations:** * Nagasaki University, Nagasaki City, Japan; † National Institute of Hygiene and Epidemiology, Hanoi, Vietnam

**Keywords:** Banna virus, molecular epidemiology, Vietnam, dispatch

## Abstract

We isolated and characterized a Banna virus from mosquitoes in Vietnam; 5 strains were isolated from field-caught mosquitoes at various locations; Banna virus was previously isolated from encephalitis patients in Yunnan, China, in 1987. Together, these findings suggest widespread distribution of this virus throughout Southeast Asia.

Banna virus (BAV) is a 12-segment, double-stranded RNA virus that is classified in the family *Reoviridae* and the genus *Seadornavirus* (*1*). BAV was first isolated from encephalitis patients in southern China, Yunnan Province, Xishuangbanna Prefecture, in 1987 (*2*). In addition, antigenetically BAV-consistent viruses were isolated from pigs, cattle, and humans in Yunnan Province and from the sera of febrile patients in Xinjiang Province (*3*). Therefore, BAV is suspected to be a pathogen of vertebrates and an encephalitis pathogen for humans. BAV was also isolated from mosquitoes in China (*3*–*5*) and Indonesia (*6*), prompting an alert about a pathogenic arbovirus in these countries. Until now, no mammalian cell line has been reported to propagate BAV, and experimental infection with BAV has not resulted in clinical encephalitis in mice (*7*). However, another species of *Seadornavirus*, the Liao Ning virus, has been propagated in various mammalian cell lines. Although this virus has caused hemorrhaging in mice, it has not been associated with the development of encephalitis (*7*).

The pathogenicity and precise distribution of *Seadornavirus* spp., including BAV, are not well understood, and the scope and impact of BAV infection in Asia require further investigation. We report the circulation of BAV in *Culex* spp. mosquitoes in Vietnam and demonstrate that 2 new phylogenetically distinct types of BAVs are co-circulating. These findings suggest that this virus is widely distributed throughout Southeast Asia.

## The Study

Mosquito samples were collected in Vietnam in 2002. Each species was separated and pooled, and each mosquito pool was then homogenized and centrifuged. The supernatant was filtrated through a 0.22-μm filter and then inoculated onto a monolayer C6/36 mosquito cell culture and subsequently incubated at 28°C for 7 days. Virus amplification in C6/36 cells was repeated twice, and the culture fluids were stored at –80°C for further analysis. RNA was extracted from the second cell culture fluid by TRIZOL LS Reagent (Invitrogen, Carlsbad, CA, USA). Reverse transcription was performed by using Superscript III Reverse Transcriptase (Invitrogen) and random hexamers, after RNA denaturation at a temperature of 95°C for 5 minutes in the presence of 15% dimethyl sulfoxide. PCR was conducted by using TaKaRa LA Taq DNA polymerase (Takara Bio, Inc., Otsu, Japan) with BAV targeting primers (for segment 5, 5′-CAGCTGCAGTGGTTATTGGA-3′ and 5′ACCGTGCATCTTAACCCTTG-3′; for segment 8, 5′-TTGCAGTCGCTGAGCTTTTA-3′ and 5′-CGCATTTGATCGTATGCTTG-3′). These targeting primers were designed from sequences of BAV strains from China and Java (GenBank accession nos. AF134519–AF134527, AY549307–AY549309, AF052024–AF052035, AY568287–AY568290, NC_004211, NC_004217-NC_004221, NC_004200-NC_004204, NC_004198, and AF052008–AF052013). The amplified cDNAs were sequenced in the 3100-Avant Genetic Analyzer (Applied Biosystems, Foster City, CA, USA). The genomic sequences of all 12 genome segments for the 5 strains were determined (GenBank accession nos. EU265673–EU265727, EU312980).

The sequences were translated to protein sequences by using the Transeq program of the EMBOSS package (*8*), version 5.00. The protein sequences were aligned with the T-COFFEE program version 5.05 (*9*) with Chinese and Javanese BAVs. Nucleotide sequences were then aligned, on the basis of protein alignment, with the Tranalign program of the EMBOSS package. Maximum likelihood phylogenetic trees were constructed from the protein-guided alignments of nucleotide sequences with DNAML software of the PHYLIP package (*10*), version 3.67.

**Figure 1:**
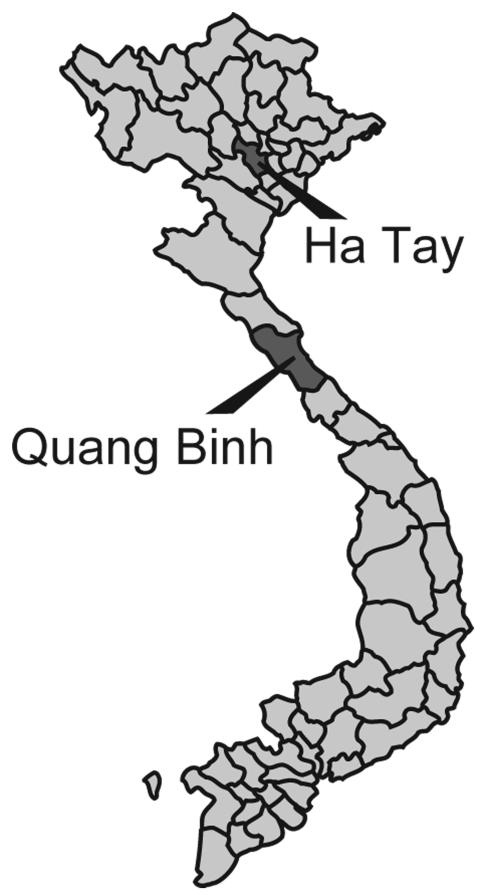
Mosquito collection sites in Vietnam.

**Figure 2:**
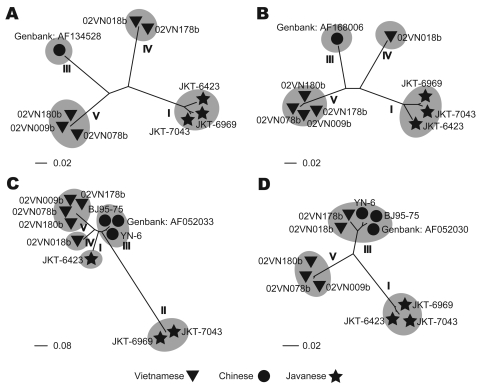
Phylogenetic trees of representative segments of Banna virus genomic RNA. A) Segment 2, encoding VP2, inner-layer coat protein. B) Segment 6, VP6, nonstructural protein, function is unknown. C) Segment 9, VP9, outer-layer attachment protein. D) Segment 12, VP12, dsRNA-binding protein. Clusters were numbered according to the clustering type classification presented in Table 2.

Five BAV strains were isolated in Ha Tay and Quang Bing Provinces (Figure 1) in Vietnam from *Culex* *annulus* and *Cx.* *tritaeniorhunchus* (Table 1). Phylogenetic analysis showed diversity within the phylogenetic clustering of each segment (Table 2). Segments 7 and 9 formed 5 independent clusters, segment 12 formed 3 clusters, and the remaining segments formed 4 clusters. Phylogenetic trees for some representative segments (segments 2, 6, 9, and 12) are shown in Figure 2. Javanese strains formed a single cluster for segments 1, 2, 6, 8, 10, and 12. For segments 7, 9, and 11, however, they diverged into 2 clusters, I and II (Figure 2, panel **C**). The Vietnamese strains were distributed within 2 independent clusters for all 12 segments**.** Segments 11 and 12 of the 2 Vietnamese strains 02VN178b and 02VN018b were included in cluster III with Chinese BAVs. Segments 6 and 9 of isolate 02VN178b belonged to cluster V, although segments 1, 2, 3, 4, and 8 belonged to cluster IV. Moreover, segments 5 and 7 of isolate 02VN178b and segments 8 and 11 of isolate 02VN009b showed a mixed electrogram pattern of clusters V and IV or III.

**Table 1:** Mosquito samples collected in Vietnam, 2002

Sample	Location	Month collected	Origin	No. mosquitoes
02VN009b	Ha Tay	Jan	*Culex annulus*	25
02VN018b	Quang Binh	Mar	*Cx. annulus*	170
02VN078b	Ha Tay	May	*Cx. tritaeniorhunchus*	100
02VN178b	Quang Binh	Aug	*Cx. tritaeniorhunchus*	102
02VN180b	Quang Binh	Aug	*Cx. tritaeniorhunchus*	83

**Table 2:** Phylogenetic clustering of Banna virus genomic RNA segments*

Strain	Isolation			Segments											
	Location	Year		1	2	3	4	5	6	7	8	9	10	11	12
Length (bp)				3747	3048	2400	2038	1716	1671	1136	1119	1101	977	867	862
Aligned area				990–3227	211–2541	108–2183	570–1748	110–760	106–1386	145–981	129–913	597–872	86–751	75–609	44–664
JKT-6423	Java, Indonesia	1980		I	I	I	I	I	I	I	I	I	I	I	I
JKT-6969	Java, Indonesia	1981		I	I	NA	NA	NA	I	II	I	II	I	II	I
JKT-7043	Java, Indonesia	1981		I	I	NA	NA	NA	I	II	I	II	I	II	I
Chinese	Yunnan, China	1987		III	III	III	III	III	III	III	III	III	III	III	III
YN-6	Yunnan, China	2000		NA	NA	NA	NA	NA	NA	NA	NA	III	NA	NA	III
BJ95-75	Beijing, China	1995		NA	NA	NA	NA	NA	NA	NA	NA	III	NA	NA	III
02VN018b	Quang Binh, Vietnam	2002		IV	IV	IV	IV	IV	IV	IV	IV	IV	IV	III	III
02VN178b	Quang Binh, Vietnam	2002		IV	IV	IV	IV	IV/V	V	IV/V	IV	V	IV	III	III
02VN009b	Ha Tay, Vietnam	2002		V	V	V	V	V	V	V	IV/V	V	V	V/ III	V
02VN078b	Ha Tay, Vietnam	2002		V	V	V	V	V	V	V	V	V	V	V	V
02VN180b	Quang Binh, Vietnam	2002		V	V	V	V	V	V	V	V	V	V	V	V

*Length, length of each segment in the reference sequence of Banna virus (BAV) from RefSeq (NC_004198, NC_004200–NC_004204, NC_004211, NC_004217–NC_004221); aligned area, the fragment used to make the alignment and phylogenetic tree. The numbers are set following the sequences of strain JHT-6423, which is the reference strain in National Center for Biotechnology Information Reference Sequences. I, the cluster including the Javanese JKT-6423 strain; II, the cluster specific to the JKT-6969 and JKT-7043 strains; III, the cluster including Chinese BAVs; IV, the cluster including the Vietnamese 02VN018b strain; V, the cluster including the Vietnamese 02VN180b strain. NA, not available.

## Conclusions

We report isolation of BAV in Vietnam. The nucleotide sequences of the Vietnamese isolates’ genomic RNA segments diverged into 2 phylogenetically distant clusters. Our data indicate that 2 BAV populations exist in the country and that both evolved independently. Two strains were clearly different from Chinese and Javanese BAVs (02VN078b and 02VN180b). Two other strains (02VN018b and 02VN178b) were phylogenetically close to Chinese BAVs when 2 shorter segments, segments 11 and 12, were analyzed but not when the remaining 10, longer segments were considered. This finding implies that a recent reassortment event has occurred and that segments of Chinese or Vietnamese strains were replaced. In addition, although some Vietnamese strains were phylogenetically distant, they could produce a viable progeny by reassortment of segments 5, 6, 7, 8, 9, and 11. This finding is clearly illustrated by the diversity in the phylogenetic clustering pattern and the mixed electrogram pattern of these segments (Table 2).

Analysis of segment 12 showed that cluster III (Table 2) contained 2 Vietnamese strains (02VN178b and 02VN018b) and the Chinese strains AF052030 and YN-6 (isolated in Yunnan Province) and BJ95-75 (isolated in Beijing). This finding suggests the possibility of wide circulation of BAVs containing segment 12 type III not only in Southeast Asia but also in East Asia.

A regionwide genotype shift of Japanese encephalitis virus (JEV), which is also carried by *Culex* spp. mosquitoes, from genotype III to genotype I, was witnessed in East Asia during the 1990s (*11*,*12*). These results and phylogenetic analysis of old JEV strains in Japan and Southeast Asia (*11*) strongly suggested that JEV could be transferred frequently from Southeast Asia to East Asia. Similarly, BAVs with segment 12 belonging to cluster type III were also distributed in both Southeast and East Asia. Therefore, we can speculate that BAVs are actively circulating within the Asian continent. To achieve a better understanding of the distribution dynamics of BAVs in Asia, more isolates are needed.

In Vietnam, a seasonal increase of viral encephalitis and meningitis cases is reported during the rainy season every year. Among these cases, 50%–70% are diagnosed as Japanese encephalitis, while the rest of them remain idiopathic (P.T. Nga et al., unpub. data). A clear association between BAV and human diseases, as well as the prevalence of BAV infection in humans, has not yet been established in Vietnam. This topic deserves further study. We also believe that BAV is a good candidate for the differential diagnosis of viral encephalitis and meningitis cases of unknown origin in tropical and subtropical Asia, where *Culex* mosquitoes are abundant.
